# 雌激素与非小细胞肺癌关系的研究进展

**DOI:** 10.3779/j.issn.1009-3419.2015.05.10

**Published:** 2015-05-20

**Affiliations:** 650118 昆明，昆明医科大学第三附属医院肿瘤生物治疗中心 Cancer Biotherapy Center, the Third Affiliated Hospital of Kunming Medical University, Kunming 650118, China

**Keywords:** 肺肿瘤, 雌激素, 雌激素受体, 抗雌激素治疗, Lung neoplasms, Estrogen, Estrogen receptors, Antiestrogens

## Abstract

肺癌死亡率位居全球癌症之首，其中约85%为非小细胞肺癌（non-small cell lung cancer, NSCLC）。虽然其治疗手段有所提高，但死亡率仍然居高不下。越来越多的证据表明，雌激素及雌激素受体参与了NSCLC的发生和发展。雌激素受体调节剂及芳香化酶抑制剂能够逆转NSCLC患者对铂类化疗药物的耐药性，并能增强表皮生长因子受体的酪氨酸酶抑制剂的疗效。本文就雌激素系统及抗雌激素治疗在NSCLC中的作用作一综述。

## Abstract (en)

Lung cancer, of which approximately 85% are non-small cell lung cancer (NSCLC), is the most leading cause of cancer mortality throughout the world. Despite recent improvements in its treatment, it still remains a highly lethal disease. It is becoming increasingly clear that estrogen and estrogen receptors are involve in the pathogenesis and development of lung cancer. Estrogen receptor modulators and aromatase inhibitor may reverse the drug-resistance of platinum based chemotherapy in NSCLC patients and can enhance curative effect of epidermal growth factor receptor tyrosine kinase inhibitor. In this article the progress in the function of estrogen in NSCLC and the treatment based on the ER signaling pathways for NSCLC is reviewed.

肺癌死亡率位居全球癌症之首，对人类健康和生命造成极大威胁^[1]^。其中非小细胞肺癌（non-small cell lung cancer, NSCLC）占所有肺癌病例的85%。虽然以手术、化疗、放疗和生物治疗等为基础的综合治疗使NSCLC在近期疗效上取得了一定进步，但其远期预后并没有得到显著改善^[2]^。因此，探寻NSCLC的发病机制和更有效的治疗手段尤为重要。大量研究证实，雌激素能够促进NSCLC细胞增殖，且雌激素受体（estrogen receptors, ER）在NSCLC中的表达情况与预后密切相关。令人兴奋的是，抗雌激素治疗可以提高NSCLC疗效、改善预后，并逐步成为NSCLC综合治疗的组成部分。本文就雌激素及其受体在NSCLC中的作用以及抗雌激素治疗在NSCLC中的应用作一综述。

## 1 雌激素与NSCLC的关系

### 1.1 雌激素促进NSCLC发生和发展

众所周知，吸烟是罹患肺癌的首要危险因素^[3]^，多项病例对照研究发现在吸烟暴露量一致的情况下，吸烟女性发生肺癌的危险性较男性高出两倍以上^[4, 5]^。而在不吸烟的人群中，女性患肺癌的风险也较男性高^[6]^。在NSCLC患者中，绝经前女性与同年龄段男性相比无生存优势，然而绝经后女性生存率却高于同年龄组男性患者^[7]^。随后，Niikawa等^[8]^发现在老年男性的NSCLC的癌组织局部雌激素浓度是绝经后女性的3.7倍。并且，在不抽烟的女性肺腺癌患者中，绝经前患者较绝经后患者的生存期更短并且肿瘤的侵袭性更强^[9]^。综合男性与女性及绝经前后女性在NSCLC的患病风险和病情进展的差异，同时考虑到男女性及绝经前后女性体内的雌激素水平差异，可以推测内源性雌激素可促进NSCLC的发生和发展。

有研究^[10, 11]^指出，雌激素与NSCLC细胞系H23共培养，可显著促进癌细胞增殖，抗雌激素药物则能阻断这种效应严重联合免疫缺陷（severe combined immune deficiency, SCID）小鼠皮下移植H23移植瘤模型以及K-ras表达和*Tp53*基因敲除小鼠肺癌模型中，雌激素能促进肿瘤生长，抗雌激素药物处理可以明显抑制肿瘤生长^[10, 11, 12]^。

### 1.2 雌激素对肺癌预后的影响

大量研究证实激素替代疗法（hormone replacement therapy, HRT）与肺癌的发生率、中位生存期及死亡率有一定的相关性。Ganti等^[13]^报道接受激素替代治疗的女性患者与未接受过HRT的女性肺癌患者相比确诊为肺癌的中位年龄明显较小，分别为63岁和68岁（*P* < 0.000, 1）。总生存期也明显缩短，分别为39个月和79个月（危险比1.97，95%CI：1.14-3.39）。妇女健康倡议（Women’s Health Initiative, WHI）以16, 608例绝经后妇女为研究对象，进行了随机对照双盲临床试验。实验对象随机分成实验组和对照组，实验组每天接受雌激素替代治疗，对照组给予安慰剂。经过5年多的激素替代治疗，实验组的肺癌相关死亡率高于对照组（*P* < 0.05），且肿瘤呈现低分化和转移倾向^[14]^。另一项由Slatore等^[15]^设计的前瞻性试验结果显示长期应用HRT，肺癌患病风险增加。

与此相反，Pesatori^[16]^对6个病例对照研究进行综合分析得出结论：HRT能够降低肺癌的发病风险。分析原因如下：雌激素对不同个体的正常支气管上皮组织和肺癌组织可能产生不同的诱导作用^[17]^；肺癌组织表达芳香化酶，可在肺部将雄烯二酮和睾酮转变为雌二醇，一部分人外源性补充雌激素后，通过负反馈调节使芳香化酶表达下降，降低了局部雌激素的浓度；雌激素的使用时间点、时长和剂量等可能影响雌激素对肺癌的作用^[18]^。

多项回顾性研究表明：抗雌激素药物的使用可以改善女性肺癌患者的预后。Bouchardy等^[19]^对6, 655例乳腺癌患者（其中有3, 066例患者使用了抗雌激素治疗）统计分析肺癌的发生率和死亡率，发现使用抗雌激素治疗的患者的肺癌死亡率显著降低。最近，加拿大学者Lother等^[20]^对2, 300例女性NSCLC患者进行了回顾性分析发现：使用抗雌激素治疗能显著降低肺癌相关死亡率。

综合分析临床前研究和临床数据可知：雌激素能促进NSCLC的发生、发展，激素替代治疗可缩短肺癌患者的生存期，抗雌激素治疗能改善肺癌预后。上述研究为抗雌激素治疗在NSCLC综合治疗中的应用提供了依据。

## 2 ER与NSCLC的关系

### 2.1 ER的分类和信号通路

ER有三个亚类，经典的核受体ER-α、ER-β和最近发现的膜相关雌激素受体G蛋白偶联的雌激素受体1（G protein-coupled estrogen receptor 1, GPER）。雌激素通过与ER结合发挥作用，典型的ER作用机制是ER-α或ER-β结合配体、同源或异源二聚化进入核内与靶基因的反式元件直接结合，或与共激活及共抑制因子结合，从而与靶基因间接结合并调控其转录，被称为基因组作用。此外雌激素还可以通过膜上的ER以及定位于胞质的ER介导，在不同的细胞发生不同的信号转导过程，即非基因组作用。非基因组作用通常会与其他多个信号通路相互作用，见图 1^[21]^。

**1:**
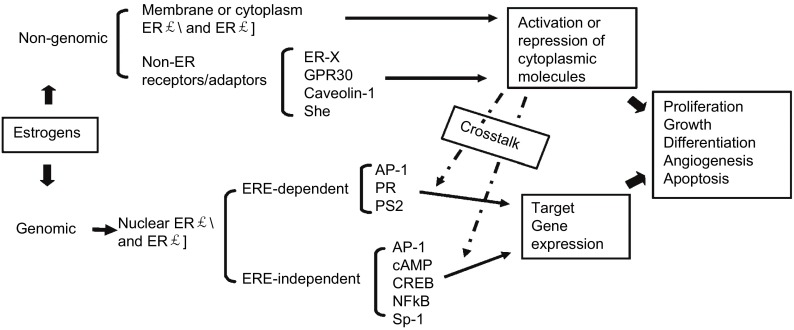
雌激素介导的信号通路。注：本图已获得版权所有者John Wiley and Sons使用许可[Chen GG, Zeng Q, Tse GM. Estrogen and its receptors in cancer. Med Res Rev, 2008, 28(6): 954-974.] Estrogen-mediated signal pathway. Note: With permission from the copyright holder John Wiley and Sons. [Chen GG, Zeng Q, Tse GM. Estrogen and its receptors in cancer. Med Res Rev, 2008, 28(6): 954-974.]

### 2.2 ER在NSCLC中的表达

随着雌激素在NSCLC中的作用逐渐被人们认知，学者们对ER在NSCLC中的表达进行探索。ER-α和ER-β在NSCLC组织中的阳性表达率各家报道结果不一，ER-α的阳性表达率在0-80%之间，ER-β的阳性表达率在9%-98%之间^[22]^。我国学者杨欣等^[23]^用698例肺癌石蜡标本进行ER-α、ER-β检测，结果显示ER-α、ER-β蛋白在正常肺组织中的阳性表达率分别为0.0%和25.0%，在肺癌组织中的阳性表达率分别为64%和72.2%。近年来发现的新的ER GPER目前在NSCLC中的研究较少，Jala等^[24]^对77例肺癌组织，8例正常肺组织和42例癌旁组织检测GPER的表达。GPER在癌组织中的阳性表达率>76%，在正常肺组织和癌旁组织的阳性表达率 < 3%。

### 2.3 ER表达与NSCLC的预后关系

越来越多的研究发现，肿瘤组织内ER的表达水平与NSCLC患者的预后存在着密切联系。Rades等^[25]^检测了64例既往接受放疗的Ⅱ期/Ⅲ期NSCLC患者ER-α、孕激素受体、雄激素受体的表达，并统计分析上述受体表达对预后的影响。结果显示：肿瘤组织表达ER-α是不良预后的危险因素，其与局部控制率差、转移、无病生存期短密切相关。日本学者Shimizu^[26]^对51例早期肺腺癌患者进行ER-α表达和表皮生长因子受体（epidermal growth factor receptor, *EGFR*）突变检测，其中有15例出现ER-α表达和*EGFR*突变的“双阳性”现象。这种“双阳性”患者无病生存期明显缩短，多因素分析显示ER-α表达和*EGFR*突变“双阳性表达”是预后较差的独立指标。ER-β有1-5五种亚型，其中ER-β1在NSCLC中研究较多^[27]^，多项研究表明，ER-β1在NSCLC中可能发挥了与ER-α不同的作用。加拿大的学者对79例NSCLC患者的研究表明ER-β1高表达无论在单因素（危险比0.41，95%CI: 0.21-0.80）还是多因素（危险比0.37，95%CI: 0.18-0.77）分析中均与较长的生存期相关^[28]^，同样的结论得到其他学者的支持^[18]^。

## 3 抗雌激素治疗与NSCLC

### 3.1 抗雌激素药物的分类及作用

抗雌激素药物主要包括选择性雌激素受体调节剂（selective estrogen receptor modulators, SERMs）、纯抗雌激素剂（pure antiestrogens）和芳香化酶抑制剂（aromatase inhibitors, AIs）三大类（表 1）。

**1:** 抗雌激素药物的分类及作用 The classification and function of anti-estrogen drugs

Class	Mechanism	Representative drugs
Selective estrogen receptor modulators	Selective estrogen receptor modulator, nonsteroidal	Tamoxifen
Pure antiestrogens	Competitively binds to estrogen receptors, antagonistic effect only	Fulvestrant
Aromatase inhibitors	Inhibits aromatase, irreversible	Exemestane Anastrozole

SERMs为ERs竞争性拮抗剂，在ERs阳性表达的肿瘤组织中，SERMs作为配体可竞争性地与ERs结合并诱导ERs发生构象变化，阻断雌二醇对ERs的激动作用从而抑制肿瘤增殖。SERMs代表性药物他莫昔芬（Tamoxifen, TAM）作为最早用于乳腺癌防治的抗雌激素药物，目前仍用于治疗ER阳性转移性乳腺癌^[29]^。纯抗雌激素剂又称为选择性雌激素受体下调剂（selective estrogen receptor downregulator, SERDs），其作用机制与SERMs不同。虽然它能竞争性与ERs结合形成复合物，但复合物不能诱导启动基因转录，而是诱导ERs快速降解，使得靶组织内ERs水平显著降低从而阻断ER信号。SERDs的代表药物是氟维司群（Fulvestrant），它无雌激素激动活性且不刺激子宫内膜增殖，目前在NSCLC中研究较多^[30]^。芳香化酶属于细胞色素P450酶系，存在于卵巢、肾上腺等很多组织中，它是雌激素合成过程中最后一步的限速酶，可将雄烯二酮和睾酮转变为雌激素酮和17β-雌二醇。芳香化酶抑制剂可以阻断芳香化酶的作用，从而降低局部雌激素的浓度，消除雌激素对肿瘤的促进作用，可用于高表达芳香化酶的NSCLC患者的综合治疗，其代表药物有依西美坦（不可逆性甾体芳香酶灭活剂）、阿那曲唑（强效非甾体类芳香化酶抑制剂）^[31]^。前两类药物可与ERs高度亲和，通过消除或干扰雌激素与ERs的相互作用促使癌细胞凋亡。后一类药物通过对细胞色素P450酶的抑制作用，减少体内雌激素水平，发挥类似抗雌激素的疗效。目前，在NSCLC中抗雌激素治疗的研究主要集中在与化疗或分子靶向药物联合使用。

### 3.2 抗雌激素治疗联合铂类化疗在NSCLC中的应用

目前以铂类药物为基础的联合化疗是NSCLC化疗的第一线标准治疗方案，然而顺铂耐药是导致NSCLC治疗失败的主要原因之一，因此寻找逆转铂类耐药的药物是肺癌治疗的研究方向之一。一项临床前试验用芳香化酶抑制剂依西美坦联合顺铂治疗NSCLC小鼠模型表明：联合组顺铂的抗肿瘤活性明显增强，移植肿瘤侵袭性明显下降^[11]^。Perez等^[32]^将高剂量的TAM联合铂类化疗，结果表明患者连续7天口服TAM，每天单剂量160 mg/m^2^即可达到逆转铂类耐药的血清浓度。Yang等^[33]^的研究表明高剂量的TAM（每天单剂量150 mg/m^2^）联合顺铂化疗，可增加患者对顺铂化疗的敏感性，并有助于改善晚期NSCLC患者的中位生存期。新一代雌激素受体竞争性拮抗剂托瑞米芬（toremifene）具有毒副作用小、耐受性好和抗肿瘤作用强等特点。Lara等^[34]^开展Ⅱ期临床研究，入组30例NSCLC患者，大剂量托瑞米芬（口服600 mg/d，连续口服7天）联合顺铂化疗，结果显示托瑞米芬在高浓度下有逆转肺癌多药耐药及化疗增敏的作用。由此可见，抗雌激素药物可以通过逆转NSCLC患者对铂类化疗耐药、增加铂类化疗的敏感性来提高肺癌疗效。

### 3.3 抗雌激素治疗联合分子靶向药物在NSCLC中的应用

近年来，NSCLC的分子靶向治疗备受关注，其中EGFR酪氨酸酶抑制剂（EGFR-tyrosine kinase inhibitor, EGFR-TKI）是NSCLC分子靶向治疗中应用最广泛的一类药物。EGFR-TKI对存在*EGFR*基因敏感突变的NSCLC患者的有效率达到75%。遗憾的是，几乎所有患者都不可避免的发生耐药，如何增强EGFR-TKI的疗效并推迟耐药时间，成为当前的研究热点^[35]^。研究^[22]^已经证实，ER和EGFR两条信号通路之间存在串扰，雌激素与ER结合后活化EGFR并引起相关信号分子的磷酸化，快速启动酪氨酸激酶旁路通过非基因途径活化EGFR促进肺癌的增殖。抗雌激素药物氟维司群联合EGFR-TKI厄洛替尼的研究，显示氟维司群能够增强厄洛替尼的抗增殖效应^[36]^。在NSCLC中，ER和胰岛素样生长因子1受体（insulin-like growth factor 1 receptor, IGFI-1R）两条信号通路之间同样存在串扰，并且雌激素能够通过活化ER-β使IGF-1R的表达上调来促进NSCLC的进展^[18]^。有学者指出，IGFI-1R信号的增强与EGFR-TKI获得性耐药有着密切关系。与吉非替尼敏感的患者相比，EGFR-TKI获得性耐药的患者的癌组织中检测到高水平的IGF-1R。EGFR-TKI治疗后，IGF-1R能够被一种异质二聚体激活，活化的IGF-1R持续向下游传递PI3K/AKT和MAPK等生存信号激活哺乳动物类雷帕霉素靶蛋白（mammalian target of rapamycin, mTOR），从而阻止细胞凋亡，这一机制参与了EGFR-TKI的获得性耐药。而在EGFR-TKI治疗的同时使用IGF-1R抑制剂，能够增强EGFR-TKI的抗增殖效应^[35]^。目前并没有证据表明雌激素及ER信号通路与EGFR-TKI耐药直接相关。基于上述研究结果，我们提出猜测：ER信号通路是否能够通过活化IGF-1R参与EGFR-TKI的获得性耐药？抗雌激素药物和IGF-1R抑制剂与EGFR-TKI联合使用，是否能进一步加强EGFR-TKI的抗肿瘤作用？

目前已开展多项抗雌激素治疗联合EGFR-TKI治疗晚期NSCLC的II期临床试验，见表 2。已经完成的氟维司群联合吉非替尼治疗绝经女性NSCLC患者和氟维司群联合厄洛替尼治疗晚期NSCLC的临床试验均显示良好的耐受性^[37, 38]^。

**2:** 氟维司群联合EGFR-TKI治疗晚期NSCLC的Ⅱ期临床试验 Ongoing phase Ⅱ clinical trials of hormonal therapy in advanced NSCLC

ClinicalTrials.gov identifier	Patient population	Preselected biomarker	Treatment
NCT01556191	Stage Ⅲ or Ⅳ NSCLC, postmenopausal women	*EGFR* mutation	Gefitinib + fulvestrant *vs* gefitinib
NCT01556191	Stage Ⅲ or Ⅳ NSCLC, postmenopausal women	*EGFR* wildtype	Erlotinib + fulvestrant *vs* erlotinib
NCT00592007	Stage Ⅲb or Ⅳ NSCLC, both gender	ER and PR expression	Erlotinib + fulvestrant
NCT00100854	Stage Ⅲb or Ⅳ NSCLC, both gender	None	Erlotinib + Fulvestrant *vs* erlotinib
NCT00932152	Stage Ⅲb or Ⅳ, postmenopausal women	None	(1) Best supportive care (2) Bevacizumab (3) Fulvestrant + anastrozole (4) Fulvestrant + anastrazole + bevacizumab
NSCLC: non-small cell lung cancer.			

## 4 展望

随着研究发现雌激素在NSCLC的发生、发展中起到促进作用，且ER在NSCLC组织中的含量、阳性表达率与肺癌的分化程度和组织学类型及预后相关，表明肺癌也是激素相关性肿瘤。已经证实拮抗雌激素治疗能够抑制肺癌细胞的生长，逆转对化疗药物的耐药性，在与化疗、分子靶向药物联合治疗NSCLC时取得一定疗效。但是，对于外源性补充雌激素和抗雌激素治疗疗效与ER表达情况的关系鲜有研究报道。NSCLC中针对雌激素的内分泌治疗是值得探索的治疗手段，深入研究雌激素及其受体促进NSCLC发生发展的分子机制，根据NSCLC表达的雌激素类型建立评价抗雌激素治疗疗效的预测指标，将为NSCLC患者个体化治疗提供新的思路。
